# The Role of Acetyl Cysteine in Cocaethylene (Non-Acetaminophen) Acute Liver Failure

**DOI:** 10.1155/2018/4393064

**Published:** 2018-09-26

**Authors:** Getaw Worku Hassen, Amaninder Dhaliwal, Catherine Ann Jenninigs, Hossein Kalantari

**Affiliations:** ^1^NYMC, Metropolitan Hospital Center, Department of Emergency Medicine, New York, NY 10029, USA; ^2^University of Nebraska, Department of Gastroenterology, Nebraska, USA; ^3^Columbia University, Postbaccalaureate Premedical Program, New York, USA

## Abstract

**Background:**

Acute liver failure can result from acetaminophen overdose, viral infection, toxins, and other disease conditions. Liver transplant is available in limited fashion and the criteria are strict as to who should get an available liver. N- Acetyl Cysteine (NAC) has been used in non-acetaminophen induced liver failure with success. Here we report a case of acute liver failure from cocaethylene that was reversed with NAC along with other medical therapy.

**Case Presentation:**

A 50-year-old female patient presented to the Emergency Department (ED) with a two-day history of coffee ground vomiting and hematemesis. She reported occasional substance abuse and heavy alcoholism. She reported shortness of breath and chest pain from the recurrent forceful vomiting. The rest of the review of systems was unremarkable except a fall from intoxication. Physical examination revealed anicteric conjunctiva and nontender abdomen and her vital signs were within normal limits. Initial blood work revealed acute liver and renal failure. The patient was started with general medical management and liver transplant service rejected the case due to active substance abuse. She underwent brief hemodialysis and was started on NAC. Over the course of her hospital stay her liver function and kidney function improved significantly and patient was discharged to home.

**Conclusion:**

In cases where liver transplant is not an option for various reasons including active substance abuse, a trial of N-Acetyl Cysteine may be beneficial and should be considered in the Emergency Department.

## 1. Introduction

 Acute liver failure can result from acetaminophen overdose, viral infection, toxins, and other disease conditions [1–3]. Liver transplant is available in limited fashion and the criteria are strict as to who should get an available liver. In case of acetaminophen induced acute liver failure N-Acetyl Cysteine (NAC) has been used with success [4]. Some case reports indicated that this treatment has also showed promising results in non-acetaminophen induced liver failure [5, 6]. Here we report a case of acute liver failure from cocaethylene (with concomitant use of alcohol and cocaine) that was reversed with NAC alongside general medical therapy, including emergent hemodialysis.

## 2. Narrative

A 50-year-old female patient presented to the Emergency Department (ED) with a two-day history of coffee ground vomiting and hematemesis. She had not been able to hold down food and was not eating much. She reported occasional cocaine abuse and heavy alcoholism. She also reported shortness of breath and chest pain from the recurrent forceful vomiting and constipation. The rest of the review of systems was unremarkable, other than a fall from intoxication. Physical examination revealed: blood pressure of 147/77 mmHg, respiratory rate of 18 breath per minute, pulse rate of 115 beats per minute, temperature of 97 degree Fahrenheit, and oxygen saturation of 100% on room air. She appeared pale and had anicteric bilateral temporal subconjunctival hemorrhage and a nontender abdomen. The rest of the physical examination was unremarkable.

Initial blood work revealed a hemoglobin/hematocrit (H/H) of 11.2mg/dL/36.2%, glucose of 35mg/dL, bicarbonate of 7 mmol/mL, and creatinine of 3.1mg/dL with an anion gap of 44. Her liver function test revealed aspartate aminotransferase (AST) 18487U/L, alanine aminotransferase (ALT) 6015U/L, alkaline phosphatase (ALK P) 229U/L, total bilirubin 2.499mg/dL, direct bilirubin 2.18mg/dL, albumin 3.3g/dL, lactate 27 mmol/L, a blood alcohol level of 36 mg/dL, and urine toxicology positive for cocaine. International Normalized Ration (INR) was 3.01 and pH was 7.06. Her acetaminophen level was 5.8mg/dL (normal range: 10-30) and the salicylate level was 3.7mg/dL (normal range: 2.8-20).

Her Model for End-Stage Liver Disease (MELD) score was 19 predicting a 3-month mortality of 6%. The patient was started with dextrose of 50%, protein pump inhibitor with medical intensive care unit (MICU), and gastrointestinal (GI) consultation. She received a PRBC transfusion along with octreotide. Liver transplant service rejected the case due to active substance abuse and, as per GI recommendation, she was started on N-Acetyl Cysteine (NAC).

An extensive work up revealed the following: Hepatitis B, C, and E were nonreactive, thyroid function was within normal limit, with slightly low complement factors 3 and 4, normal myeloperoxidase antibody, normal copper level, slightly elevated CA19, and Alpha Fetoprotein (AFP). Proteinase-3 A and Alpha 1 Antitrypsin negative were negative, and Ceruloplasmin was slightly decreased. The patient had slightly elevated iron level, slightly decreased Tissue Iron Binding Capacity (TIBC), and transferrin saturation of 76%. Tests for mitochondrial antibody, smooth muscle antibody negative, and liver kidney microsomal antibody were negative, and there was low Factor V level. Normal antinuclear and Anti-Streptolysin antibody were normal. No assay was performed to detect cocaethylene as it is not a routine test.

The patient received general medical stabilization and treatment, including brief hemodialysis and treatment with NAC over the course of her hospital stay, and experienced significant improvement and near resolution of both liver and renal function abnormalities. At discharge her laboratory values were as follows: AST 64 U/L, ALT 216 U/L, ALP 142 U/L, creatinine 1.7mg/dL, total bilirubin 1.23mmol/L, direct bilirubin 0.79mmol/L, INR 1.26, H/H 11.5mg/dL/35.6%, ammonia 25Umol/L, and lactate 1.8mmol/L.

The patient was discharged to her home in improved condition and advised to follow up in the outpatient clinic with counseling for alcohol and substance abuse.

## 3. Discussion

Acetaminophen is the major cause of acute liver failure, but other factors such as viral infection, chronic alcohol abuse, and other toxins and drugs, as well as some medical conditions, lead to liver failure [1–3, 7, 8]. A combination of any of the abovementioned causes poses a greater risk for liver damage. The toxic effects of the cocaine-alcohol combination have been observed both in Emergency Department (ED) and in inpatient medical units. Ethanol alters the hepatic biotransformation of cocaine, resulting in a novel active metabolite, cocaethylene [9–11]. Oral cocaine was suggested to produce relatively larger concentrations of cocaethylene [12–15]. Drug abusers combine cocaine and alcohol to get intense high and less paranoia when coming off the high [16–18]. Both alcohol and cocaine cause liver toxicity, but cocaethylene appears to be more potent in its toxicity than cocaine [19–22].

Of the various mechanisms of N-Acetyl-P Aminophenol (APAP), acetaminophen, and metabolism approximately 5% to 10% are metabolized by cytochrome P450 system to N-acetyl-p-benzoquinoneimine (NAPQI) [23], a highly reactive molecule that damages hepatocytes by formation of covalent bonds with other intracellular proteins. This reaction is prevented by conjugation with glutathione and subsequent reactions to generate a water-soluble product [24, 25]. Overdose acetaminophen is increasingly metabolized by cytochrome P450 generating NAPQI in amounts that can deplete glutathione. With glutathione depletion and not being replenished faster, NAPQI will begin to accumulate in the hepatocytes resulting in direct hepatocyte damage [3].

Glutathione is important in preventing the accumulation of peroxides and superoxide radicals and is reduced from 70% to 80% in cases of acetaminophen induced liver cell toxicity [26]. There is approximately 20%-40% reduction in glutathione with cocaine-induced liver cell necrosis [27, 28]. On the contrary, overnight fasting causes a 50% reduction in glutathione without associated liver cell damage [29] and glutathione decrease in itself does not explain covalent binding of metabolically active cocaine metabolites to hepatic proteins [30]. These factors imply that there must be other reasons for liver toxicity other than glutathione depletion. N-acetyl cysteine (NAC), a precursor of glutathione, is an effective antidote for APAP poisoning. When administered early after an acute APAP overdose, NAC provides cysteine for the replenishment and maintenance of hepatic glutathione stores, enhances the sulfation pathway of elimination, and may directly reduce NAPQI back to acetaminophen [31, 32]. Some work showed improved transplant-free survival in patients with APAP-induced fulminant liver failure (FHF) [33, 34]. The mechanism here is not the detoxification of NAPQI but rather enhanced recovery. Several different mechanisms seem to contribute to the efficacy of NAC in this setting. NAC improves hepatic perfusion and oxygen delivery and extraction in patients with APAP-induced FHF [35]. Other beneficial effects include scavenging of reactive oxygen and nitrogen species and improved mitochondrial energy production [36, 37]. These beneficial effects of NAC do not seem to be unique to APAP hepatotoxicity [5].

The mechanisms by which cocaine causes liver cell injury appear to be similar to that of liver injury caused by the toxic metabolite of acetaminophen. It is related to production of highly reactive metabolites, with peroxidation, free radical formation, and covalent binding to hepatic proteins. Approximately 10% of cocaine undergoes N-demethylation in hepatocytes by the cytochrome P-450-mixed function oxidase system, forming norcocaine, a metabolite that elicits significant liver cell damage when injected intraperitoneally in mice [13–15]. This is due to further enzymatic breakdown to N-hydroxynorcocaine and norcocaine nitroxide [38]. Oxidation to the nitrosonium ion showed the latter to be highly reactive with glutathione, serving as catalyst for the conversion of alcohols, amines, and hydroxide ions to aldehydes, ketones, and hydrogen peroxide and causing lipid peroxidation of cell membranes [12, 39–41].

Patients with acute liver failure (ALF) could deteriorate rapidly, and although a minority of patients may recover, the majority require liver transplantation as a life-saving therapy. Patients need to be immediately recognized, optimizing treatment started, and centers for liver transplantation contacted to facilitate the process. Before the era of transplantation, the mortality rate from ALF was greater than 80% [42]. Survival rates have improved significantly with better understanding of the clinical syndrome, earlier recognition, intensive care monitoring, and transplantation [43].

Compared to patients with acetaminophen induced ALF, patient with non-acetaminophen induced ALF have a spontaneous survival rate of 30%. Patients with non-acetaminophen toxicity have limited therapeutic options and the majority of them require transplantation [43, 44]. Unfortunately, there are strict criteria to qualify for liver transplantation and active substance abuse makes a candidate less fit to get transplant. Given the limited therapeutic options and the graveness of the disease, attempt have been made to treat patients with non-acetaminophen ALF using NAC. Few case reports have shown promising results, especially when applied early on in patents with lower coma grades [6, 45–47]. The first prospective, double-blinded, randomized control study with NAC in non-acetaminophen induced liver failure showed a significant improvement in patients with lower-grade encephalopathy. The transplant-free survival for NAC group was higher (40%) than those without NAC (27%) [5].

In summary, even though both alcohol and cocaine can lead to liver toxicity, the acute liver failure is presumed to be from the potent effect of cocaethylen even if no assay was performed to quantify the cocaethylene level. A potential toxic agent is acetaminophen, but with no toxic blood level it is less likely to be the cause of the acute liver failure. N-acetylcysteine offers potential and early treatment options for patients with ALF from non-acetaminophen causes to improve liver function rapidly and in severe cases until transplantation is available.

## 4. Conclusion

Liver failure is the result of multiple pathological conditions, toxin effects, and viral infection. N-acetylcysteine has been predominantly used for acetaminophen induced liver failure, in some instance, in non-acetaminophen induced liver failure, but it has also had some success in some instance of non-acetaminophen induced liver failure. In cases where liver transplant is not an option for various reasons, including active substance abuse as in this patient's case, a trial of NAC may be beneficial.
